# Carbohydrate-Rich Extract from *Pereskia aculeata* Leaves: *In Vitro* Prebiotic-Related Properties and Metabolic Effects in an Experimental Model of Obesity

**DOI:** 10.1007/s11130-026-01486-0

**Published:** 2026-03-21

**Authors:** Martha Eunice de Bessa, Vivian Tomasco Andrade, Gabriele Moreira Guimarães, Ana Flávia Lawall Werneck Cerqueira, Carolina Carvalho Ramos Viana, Marianna Miranda Furtado, Renata de Freitas Mendes, Mirian Pereira Rodarte, Anderson S. Sant’Ana, Maria José Valenzuela Bell, Maria Christina Marques Nogueira Castañon, Maria Silvana Alves, Elisabeth Neumann, Elita Scio

**Affiliations:** 1 Laboratory of Bioactive Natural Products, Department of Biochemistry, Biological Sciences Institute, Federal University of Juiz de Fora, Juiz de Fora, 36.036-900 MG Brazil; 2 Department of Microbiology, Biological Sciences Institute, Federal University of Minas Gerais, Belo Horizonte, Minas Gerais 31270-901 Brazil; 3 Department of Pharmaceutical Sciences, Faculty of Pharmacy, Federal University of Juiz de Fora, Juiz de Fora, MG 36.036-900 Brazil; 4 Department of Physics, Institute of Exact Sciences, Federal University of Juiz de Fora, Juiz de Fora, MG 36036-900 Brazil; 5 Cândido Tostes Dairy Institute, Juiz de Fora, MG 36045-560 Brazil; 6 Department of Food Science and Nutrition, Faculty of Food Engineering, University of Campinas, Campinas, SP 13083-862 Brazil; 7 Department of Morphology, Institute of Biological Sciences, Federal University of Juiz de Fora, Juiz de Fora, MG 36036-900 Brazil

**Keywords:** *Pereskia aculeata*, Soluble carbohydrates, Non-starch polysaccharides, Prebiotic-related potential, Metabolic health, Obesity

## Abstract

*Pereskia aculeata* Mill. is a leafy cactus native to Brazil, whose leaves are traditionally used as food and are characterized by high nutritional value and significant mucilage content. This study aimed to evaluate the functional potential of the carbohydrate-rich extract obtained from leaves and to investigate its metabolic effects in obese male rats subjected to a litter-size reduction model. The extract was chemically characterized by physicochemical analysis and FTIR-MIR spectroscopy, which revealed spectral features consistent with arabinogalactan-type carbohydrates (absorption bands between 1,000 and 1,050 cm⁻¹). In vitro assays demonstrated antioxidant activity, with DPPH radical inhibition exceeding 60% and nitric oxide reduction exceeding 30%. In addition to promoting the growth of lactic acid bacteria in culture medium supplemented with the extract, it also showed antagonistic activity against selected Gram-negative pathogens. FTIR-MIR analysis during an in vitro gastrointestinal simulation indicated relative spectral stability. In vivo, obese rats were supplemented with the extract for 75 days (0.3%, w/v, in drinking water; 300 mg/kg body weight/day). Supplemented animals showed a reduction in visceral adiposity (-20%) and serum triglycerides (-31%) compared to obese controls (*p* < 0.05). The obesity model increased fasting blood glucose by approximately 19%, and supplementation reduced blood glucose by approximately 10%. Data were analyzed using appropriate parametric tests (*p* < 0.05). Liver parameters and histological analyses indicated protection against non-alcoholic fatty liver disease. Overall, these results corroborate the functional potential of carbohydrate fractions and provide a basis for future studies addressing microbiota-mediated mechanisms and metabolic effects.

**Supplementary Information:**

The online version contains supplementary material available at 10.1007/s11130-026-01486-0.

## Introduction

*Pereskia aculeata* Mill., popularly known as ora-pro-nóbis (OPN), is a native but non-endemic species widely distributed from northern to southern Brazil [1]. It differs from most Cactaceae species by having true, fleshy leaves, which are traditionally consumed as food in specific regions of the country. OPN leaves contain a high concentration of bioactive compounds, including essential vitamins, minerals, and non-essential amino acids, contributing to their nutritional and functional value [2]. The leaves are considered non-toxic and represent a source of hemicelluloses and mucilage-type dietary fibers, which are predominantly soluble [3]. Dietary fibers from different plant sources, particularly those with low digestibility, are widely known to interact with the intestinal microbiome. These compounds play a fundamental role in maintaining digestive health and have been associated with metabolic benefits, including obesity and glycemic regulation [4, 5]. Given the relationship between gut microbiota and metabolic health, dietary modulation has been investigated as a strategy to prevent dysbiosis-related conditions [6–9]. Plant-derived carbohydrates with limited digestibility have received increasing attention for their functional potential. Such ingredients may contribute to nutritional strategies aimed at the prevention and management of metabolic disorders [10].

In this context, *Pereskia aculeata* Mill emerges as a promising plant source of complex carbohydrates with potential functional relevance. Considering its traditional consumption, nutritional profile, and fiber composition, the present study aimed to obtain and characterize a carbohydrate-rich extract from *P. aculeata* leaves and evaluate its antioxidant and prebiotic potential under in vitro conditions, as well as its effects on metabolic parameters related to obesity.

## Materials and Methods

Detailed procedures are provided in Supplementary Methods.

### Plant Material and Extract Preparation

A carbohydrate-rich extract was obtained from the leaves of *P. aculeata*, collected in Juiz de Fora, Minas Gerais, Brazil (21°40′04.0″ S; 43°17′44.1″ W). A voucher specimen (CESJ No. 57539) was deposited at the Leopoldo Krieger Herbarium of the Federal University of Juiz de Fora (UFJF), and access to genetic heritage was registered in SISGEN (ACAC564).

### Proximate Composition and Phytochemical Screening

The proximate composition of leaf powder and carbohydrate-rich extract was determined using standard food analysis procedures [11], including Soxhlet extraction for lipids and the Kjeldahl method for proteins. Moisture and ash were determined gravimetrically; total carbohydrates were determined by difference; and reducing sugars were determined according to Nelson [12]. Preliminary phytochemical screening of the main classes of secondary metabolites was performed following the procedures described by Matos (1997) [13]. Analyses were performed in triplicate.

### FTIR-MIR Spectroscopic Characterization

Structural characterization of the extract was performed by Fourier-transform mid-infrared spectroscopy (FTIR-MIR) [14]. Spectra were recorded using OPUS^®^ software (version 6.5) in the range of 400–4,000 cm⁻¹, with 64 scans. Inulin, a well-established prebiotic oligosaccharide, was used as a reference material for comparative purposes.

### *In vitro* Antioxidant Activity

Free radical scavenging activity was assessed using the DPPH assay as described by Bondet et al. [15], the nitric oxide (NO) scavenging activity was determined using sodium nitroprusside as an NO donor, following the method of Green et al. [16], and total antioxidant capacity was evaluated using the phosphomolybdenum reduction assay, as described by Prieto et al. [17].

### Evaluation of Prebiotic Potential

The prebiotic potential of the carbohydrate-rich extract was assessed based on its ability to support the growth of selected lactic acid bacteria (LAB), inhibit pathogenic microorganisms, and withstand simulated gastrointestinal digestion [18]. LAB growth was evaluated in a modified MRS medium in which dextrose was replaced with the carbohydrate-rich extract, and bacterial proliferation was monitored by viable cell counting and optical density measurements. Antagonistic activity against selected pathogens was assessed using a spot-on-lawn assay [19]. Simulated gastrointestinal digestion was performed according to the standardized INFOGEST protocol [20], and FTIR-MIR was used to analyze samples collected at each phase to assess structural stability.

## Experimental Animals and Obesity Model

Male Wistar rats (21 days old, 25–35 g) were obtained from the Center for Reproductive Biology (UFJF) and maintained with free access to water and a commercial standard diet (Nuvital^®^TM, Brazil). All procedures were approved by the Institutional Ethics Committee (protocol No. 01/2018). Obesity was induced using the litter-size reduction model described by Rinaldi et al. [21]. Animals were divided into three groups (*n* = 10): normal litter (GN), reduced litter control (GC), and reduced litter supplemented with the carbohydrate-rich extract (T1). The T1 group received the extract in drinking water (0.3% w/v, ad libitum) for 75 days (~ 300 mg/kg body weight/day), a dose defined based on concentrations commonly used for inulin in experimental prebiotic studies [22]. At the end of the protocol, animals were euthanized by intraperitoneal administration of ketamine (90 mg/kg) and xylazine (10 mg/kg), and the liver, retroperitoneal adipose tissue, and perigonadal adipose tissues were collected for analysis.

### Biochemical and Histopathological Analyses

Oral glucose tolerance test (OGTT) was performed at 96 days of age after a 6-hour fasting period by oral administration of glucose (2 g/kg body weight). Blood glucose was measured from tail tip blood samples at baseline and at 30, 60, 90, and 120 min using a portable glucometer (Accu-Chek Active^®^, Roche^®^). Serum biochemical parameters (glucose, creatinine, ALT, AST, total cholesterol, LDL, HDL, and triglycerides) were determined using commercial diagnostic kits (Roche^®^) compatible with the Cobas C111 automated analyzer.

For histopathological analysis, liver samples were fixed in 10% formalin, embedded in paraffin, sectioned at 4 μm thickness, and stained with hematoxylin and eosin. Steatosis was graded by two independent observers according to established criteria [23], based on the percentage of hepatic parenchyma affected.

### Statistical Analysis

Data are expressed as mean ± standard deviation (SD). For comparisons among three groups, data were analyzed by one-way analysis of variance (ANOVA), followed by Tukey’s *post hoc* test. For comparisons between two independent groups or matrices (*e.g*., leaf powder vs. carbohydrate-rich extract, or enriched vs. standard culture media at the same time point), Student’s *t*-test (independent samples) was applied.

Normality was assessed using the Shapiro–Wilk test, and homogeneity of variances was evaluated using Brown–Forsythe and Bartlett’s tests prior to parametric analysis. Differences were considered statistically significant at *p* < 0.05. Statistical analyses were performed using GraphPad Prism^®^ version 7.0 (GraphPad Software, San Diego, CA, USA).

## Results and Discussion

Table 1, available in the supplementary material, summarizes the physicochemical parameters of dried *P. aculeata* leaves and the carbohydrate-rich extract obtained from the leaves. The carbohydrate-rich extract showed a markedly higher carbohydrate content (approximately 90%) compared with the dried leaf powder (53%). Phytochemical screening did not detect any secondary metabolites. The overall extraction yield was 9.8% based on dried plant material. These results indicate that the extraction procedure effectively enriched the carbohydrate fraction, consistent with the later structural profile observed by FTIR-MIR analysis.

### FTIR-MIR Spectroscopic Analysis

**Fig. 1:**
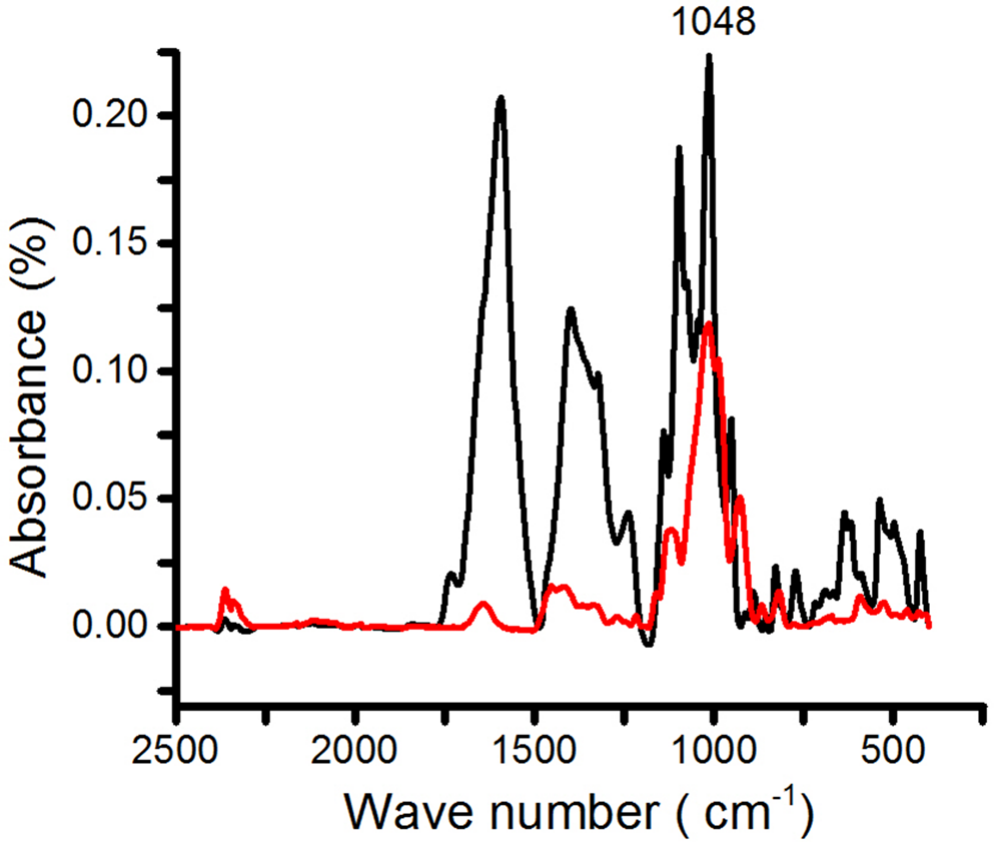
FTIR-MIR spectra of the carbohydrate-rich extract (black line) and inulin (I) (red line)

The absorption bands observed between 1,000 and 1,050 cm⁻¹ in Fig. 1 are characteristic of carbohydrate structures commonly associated with polysaccharides reported for *P. aculeata*, including spectral features consistent with arabinogalactan-type carbohydrates. In addition, intense absorption bands in the region between 1,150 and 1,000 cm⁻¹ (Fig. 1) were attributed to overlapping ring vibrations, stretching of lateral hydroxyl groups (C–OH), and O-glycosidic linkages (C–O–C), while the broad band between 1,700 and 1,500 cm⁻¹ is related to amide functional groups (RCONHR) [24]. Previous studies by Martin et al. [25] demonstrated that polysaccharides from *P. aculeata* are mainly composed of *β*-D-galactose units linked through (1→4) bonds, characterizing type I arabinogalactans. Although no direct structural determination was performed in the present study, the spectral profile observed is consistent with these previously described carbohydrate features. Non-starch polysaccharides such as arabinogalactans have been associated with biological effects that depend on botanical source and structural characteristics, including immunomodulatory properties [26]. Absorption bands near 1048 cm⁻¹ were also observed in Fig. 1, supporting the presence of carbohydrate structures with spectral profiles qualitatively comparable to those of inulin, which was used as a reference prebiotic in this study [18, 27].

### Antioxidant Activity of the Carbohydrate-rich Extract

**Fig. 2:**
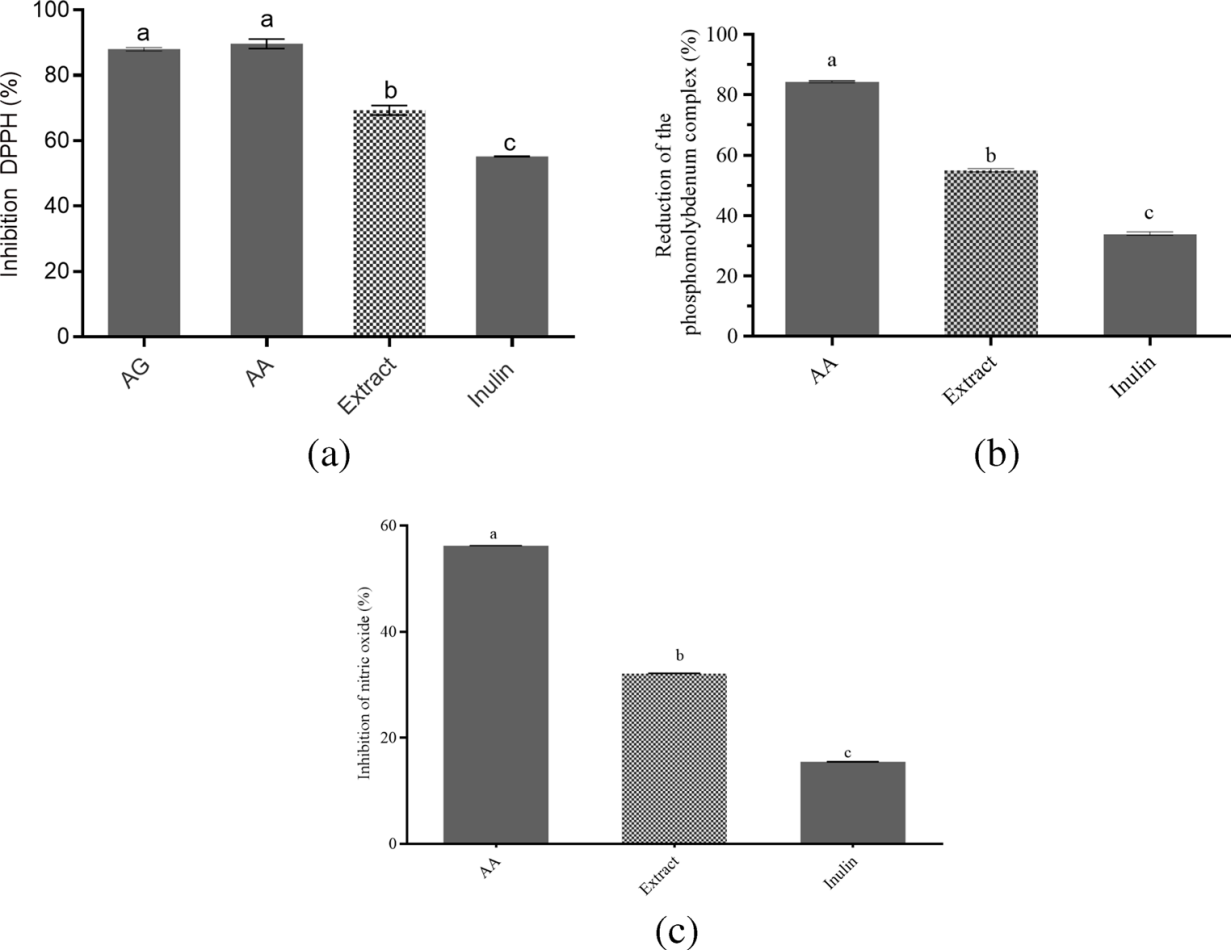
Antioxidant activity of the carbohydrate-rich extract evaluated by different in vitro assays. (**a**) DPPH radical scavenging activity. (**b**) Phosphomolybdenum complex reduction assay. (**c**) Inhibition of nitric oxide (NO) production. AG, gallic acid; AA, ascorbic acid; Extract, carbohydrate-rich extract; Inulin. Bars represent mean ± SD. Different lowercase letters indicate statistically significant differences between groups (*p* < 0.05)

The antioxidant activity of the carbohydrate-rich extract was evaluated using complementary in vitro assays, including DPPH radical scavenging, phosphomolybdenum complex reduction, and inhibition of nitric oxide (NO) production (Fig. 2a–c). The extract exhibited radical scavenging activity above 60% in the DPPH assay (Fig. 2a) and reduced the phosphomolybdenum complex by more than 50%, indicating appreciable total antioxidant capacity (Fig. 2b). Nitric oxide inhibition results are shown in Fig. 2c, in which the carbohydrate-rich extract, at 125 µg/mL, showed higher NO inhibitory activity than inulin at the same concentration, indicating a greater NO scavenging effect. These findings support the functional potential of the carbohydrate-rich extract as a food ingredient, in agreement with reports on carbohydrate-rich plant-derived fractions [26, 27].

### Prebiotic Potential of the Carbohydrate-rich Extract

#### Growth of Lactic Acid Bacteria

Prebiotics are defined as substrates that selectively stimulate the growth and activity of beneficial microorganisms, such as lactic acid bacteria (LAB), which play an essential role in intestinal health [28]. Viable cell counts of different LAB strains grown on standard MRS agar (M) and on MRS agar supplemented with the carbohydrate-rich extract (E) are shown in Table 2 - available in the supplementary material. Group 1 included *Lactobacillus acidophilus* NCFM (EA/MA), *Lacticaseibacillus casei* 25P (EC/MC), and *Lactobacillus delbrueckii* UFV-H2-b20 (EDe/MDe). In contrast, Group 2 included *Limosilactobacillus fermentum* ATCC 14,931 (EF/MF), *Limosilactobacillus reuteri* 1/2Z (ER/MR), and *Lactiplantibacillus plantarum* ATCC 14,917 (EP/MP). The growth of LAB strains was significantly higher in MRS medium supplemented with the carbohydrate-rich extract compared with standard MRS medium. Except for *Lacticaseibacillus casei* 25P, all strains exhibited significantly increased growth in the extract-enriched medium from 4 h of incubation onward. These results indicate that the carbohydrate-rich extract acted as an effective fermentable substrate, supporting LAB growth in vitro, in agreement with characteristics commonly attributed to prebiotic carbohydrate fractions.

#### Antagonistic Activity against Pathogenic Microorganisms

The ability of lactic acid bacteria (LAB) to inhibit pathogenic microorganisms is commonly attributed to antimicrobial metabolites produced during fermentation, such as organic acids and other inhibitory compounds [28, 29]. In addition, prebiotic compounds may indirectly influence pathogen control by favoring the growth and metabolic activity of beneficial microorganisms [19, 27]. The fermentation of prebiotic carbohydrates by lactic acid bacteria produces metabolites, particularly organic acids, that lower environmental pH and exert antimicrobial effects against potential pathogens. In our study, the inhibitory activity observed against potential pathogenic bacteria is consistent with this mechanism and supports the functional relevance of the carbohydrate-rich extract. Although systemic effects such as reduced metabolic endotoxemia, attenuation of inflammation, and improved insulin sensitivity associated with prebiotic intake were not directly evaluated in the present study [30], modulation of LAB activity may be relevant within this broader context. In the present study, LAB cultivated in MRS medium supplemented with the carbohydrate-rich extract produced metabolites that resulted in greater inhibition of *Salmonella enterica* subsp. enterica serovar *Typhimurium* and *Escherichia coli* compared with LAB cultivated in standard MRS medium (Table 3 - available in the supplementary material). The inhibition halos observed for LAB grown in extract-enriched MRS medium were significantly larger than those observed for LAB grown in standard MRS medium (*p* < 0.05), indicating increased antagonistic activity under the experimental conditions evaluated.

#### Digestibility of Carbohydrate-rich Extract

**Fig. 3:**
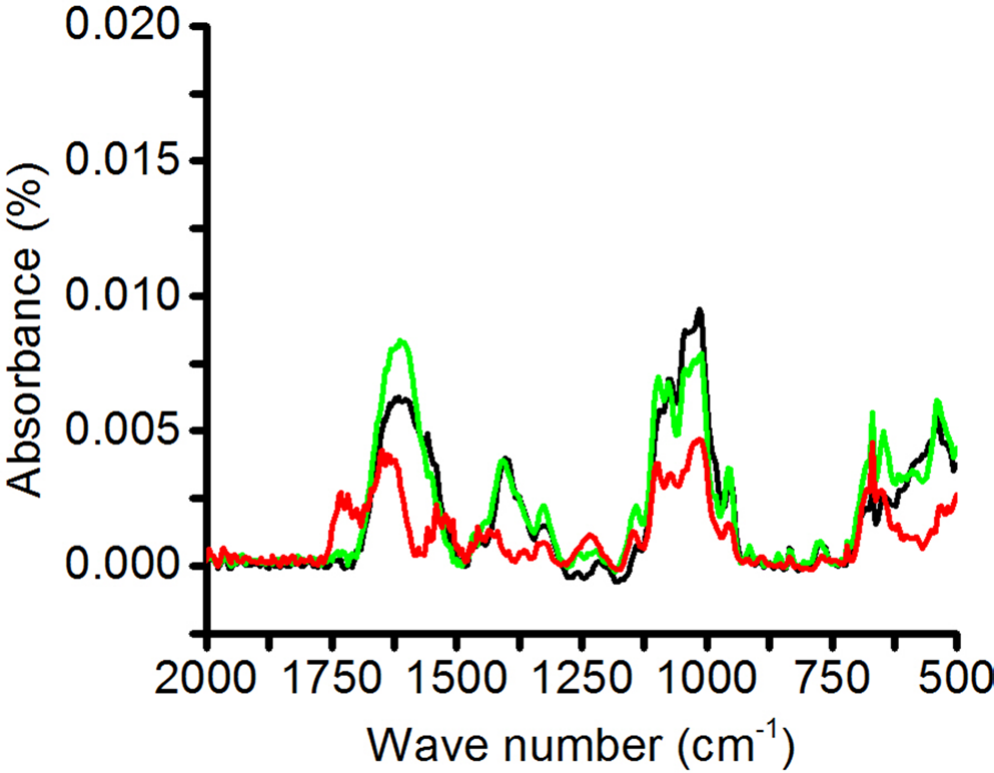
FTIR-MIR spectra of the carbohydrate-rich extract obtained at different stages of the in vitro gastrointestinal digestion simulation. Black, green, and red lines correspond to jejunal, duodenal, and gastric phases, respectively

The FTIR-MIR spectral profiles obtained at different stages of the in vitro gastrointestinal simulation showed that the carbohydrate-rich extract did not undergo significant changes in its spectral profile (Fig. 3). Variations in band intensity were attributed to differences in sample concentration at each stage, and not to shifts in characteristic absorption bands. Although definitive structural preservation cannot be guaranteed, the relative spectral stability observed during simulated upper gastrointestinal digestion suggests limited digestibility of the extract, a fundamental characteristic of prebiotic substrates, since poorly digestible carbohydrates can reach the colon and serve as fermentable substrates for intestinal bacteria [29]. Taken together, the relative spectral stability of the extract during digestion, the in vitro promotion of lactic acid bacteria growth, and the antagonism on potential pathogens suggest that the extract can be classified as a carbohydrate fraction with prebiotic functional potential and provide a basis for investigating its metabolic effects in an experimental model of obesity.

### Effects of the Carbohydrate-rich Extract on Obesity-related Alterations

#### Obesity Control

The Lee index is commonly used to identify obesity in experimental animals; values above 0.30 indicate excess body fat. At the same time, body mass index (BMI) can also be applied to assess adiposity in rats (Table 4) [31]. As shown in Table 4 (available in the supplementary material), animals in the obese control group (GC) exhibited significantly higher BMI and Lee index values than those in the normal group (GN). In contrast, animals supplemented with the carbohydrate-rich extract (T1) showed significantly lower BMI values than those in the GC, remaining below the obesity threshold, indicating attenuation of obesity-related anthropometric parameters under the experimental conditions tested. Consistently, obesity-related markers listed in Table 5 (available in the supplementary material) were significantly lower in both GN and T1 groups compared with GC. Although values in the T1 group remained higher than those observed in GN, they were markedly lower than those in GC, indicating partial attenuation of excessive fat accumulation.

**Fig. 4:**
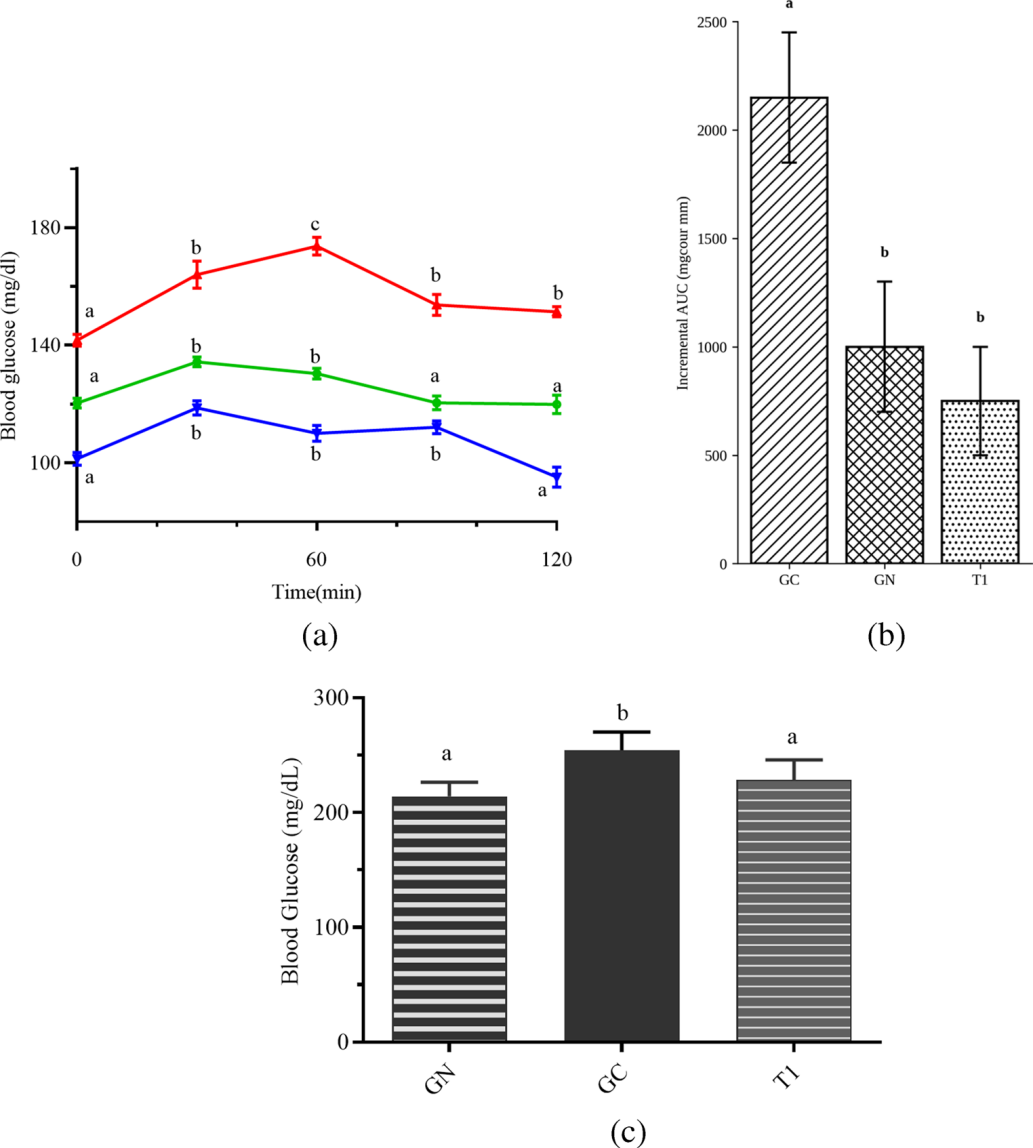
Effects of the carbohydrate-rich extract on glucose homeostasis. GN, normal group; GC, obese control group; T1, group supplemented with a carbohydrate-rich extract. (**a**) Oral glucose tolerance test (OGTT). Different lowercase letters along the same line indicate statistically significant differences in blood glucose levels over time within the same group (*p* < 0.05). (**b**) Incremental area under the curve (iAUC) calculated by the trapezoidal method, subtracting fasting glycemia (time 0) from subsequent time points. (**c**) Fasting blood glucose levels measured after 6 h of fasting. Bars represent mean ± SD. Different lowercase letters indicate statistically significant differences between groups (*p* < 0.05)

Following glucose overload, GC exhibited impaired glucose tolerance, with blood glucose levels exceeding 140 mg/dL at multiple time points during the oral glucose tolerance test, whereas T1 maintained blood glucose levels below this threshold throughout the test (Fig. 4a). In agreement with these findings, fasting blood glucose levels were significantly higher in GC compared with GN, while treatment with the carbohydrate-rich extract reduced fasting glycemia to levels comparable to those observed in GN (Fig. 4b).

#### Lipid Profile

Obese individuals tend to extract more energy from their diet and consequently accumulate greater body fat [32]. The lipid profile data obtained in the present study corroborate this concept and are summarized in Table 5, available in the supplementary material. Supplementation with the carbohydrate-rich extract (T1) was associated with improvements in lipid profile parameters, with total cholesterol, LDL cholesterol, triglycerides, and HDL cholesterol values remaining closer to those observed in the normal group. These findings indicate attenuation of obesity-associated dyslipidemia in animals receiving the extract. Obesity-induced inflammation is closely linked to visceral fat accumulation and excess body weight, and it tends to decrease with reductions in adiposity [33]. Similar effects were reported by Everard et al. [34], who demonstrated reduced hyperlipidemia following the intake of prebiotic-enriched diets. Elevated triglycerides are the main metabolic determinant of hepatic fat accumulation. The combination of elevated triglycerides and increased LDL is the most common biochemical profile observed in hepatic steatosis. The significant reduction in serum triglycerides observed in the supplemented group reinforces the potential protective effect of the extract against the development of obesity-related fatty liver.

#### Hepatic Function

**Fig. 5:**
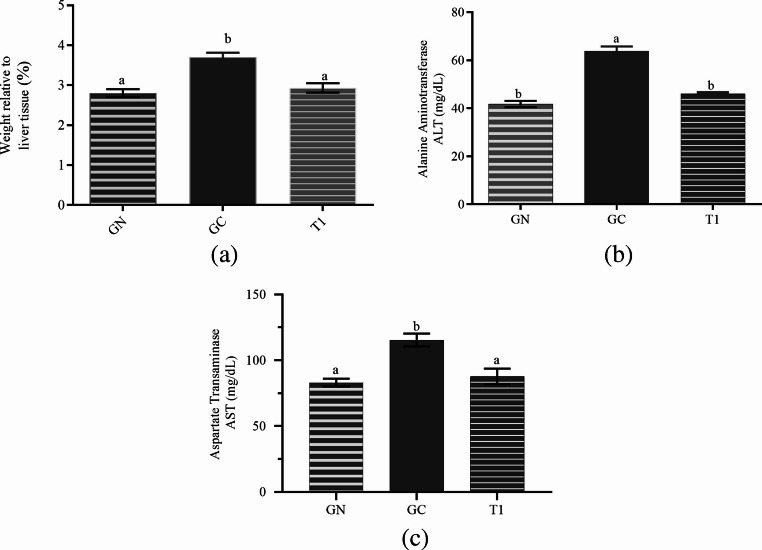
Effect of supplementation with the carbohydrate-rich extract on relative liver weight, and on serum liver enzymes in Wistar rats. (**a**) Relative liver weight. GN, normal group; GC, obese control group; T1, group supplemented with a carbohydrate-rich extract. Bars represent mean ± SD. Different superscript lowercase letters indicate statistically significant differences between groups (*p* < 0.05). Serum liver enzymes (**b**) Alanine aminotransferase (ALT) and (**c**) Aspartate aminotransferase (AST) levels in Wistar rats. GN, normal group; GC, obese control group; T1, group supplemented with the carbohydrate-rich extract. Bars represent mean ± SD. Different superscript lowercase letters indicate statistically significant differences between groups (*p* < 0.05)

Hepatic function was evaluated by alanine aminotransferase (ALT) and aspartate aminotransferase (AST) activities. Hypercholesterolemia and elevated triglyceride levels are strongly associated with non-alcoholic fatty liver disease, which encompasses pathological alterations such as hepatic steatosis and non-alcoholic steatohepatitis [35]. As shown in Fig. 5a, the obese control group (GC) exhibited a significantly higher relative liver weight than the normal group (GN), a finding commonly associated with hepatic lipid accumulation in obesity-related conditions. In contrast, animals supplemented with the carbohydrate-rich extract (T1) showed a significant reduction in relative liver weight compared with GC, with values remaining closer to those observed in GN, indicating attenuation of obesity-related hepatic alterations. Hepatic steatosis is frequently accompanied by elevated serum AST and ALT levels, which are commonly used biomarkers of liver injury. As shown in Fig. 5b and c, the obese control group displayed significantly higher transaminase levels than the GN group. In contrast, supplementation with the carbohydrate-rich extract significantly reduced ALT and AST levels compared with GC, with enzyme activities remaining comparable to those in the normal group. These findings suggest an association between extract supplementation and improved liver-related biochemical parameters, consistent with previous reports linking metabolic improvements to reduced hepatic stress [35].

#### Histopathological Alterations in Hepatic Tissue

**Fig. 6:**
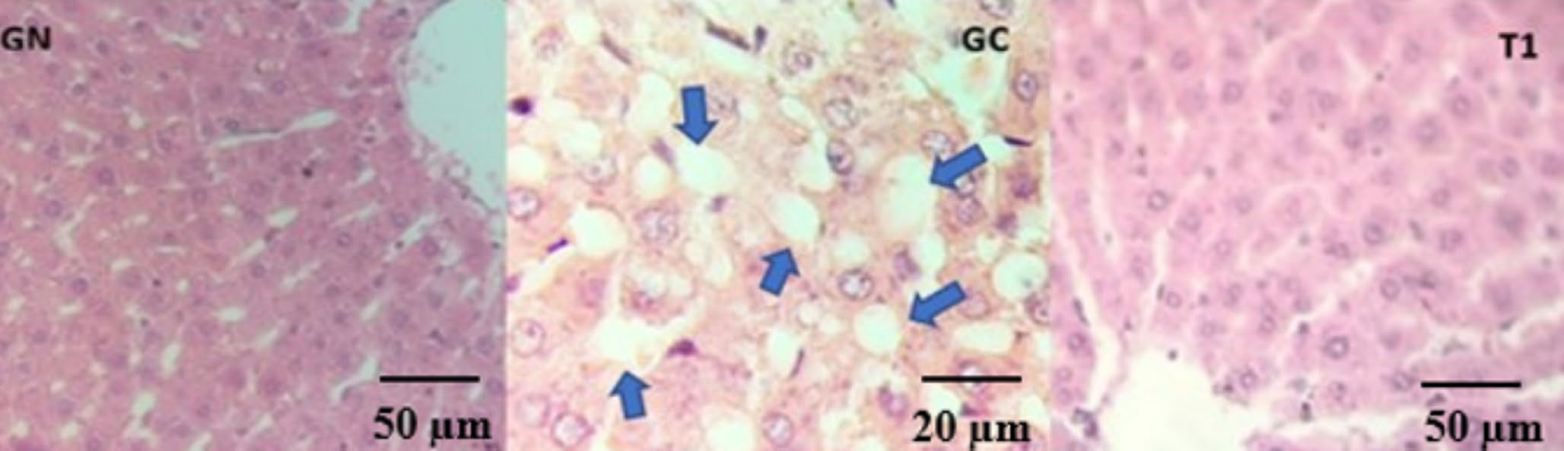
Representative histological sections of liver tissue from GN (normal group), GC (obese control group), and T1 (group supplemented with the carbohydrate-rich extract). Blue arrows indicate lipid droplets characteristic of hepatic steatosis (Hematoxylin and eosin staining; original magnification GN, T1 × 100; and GC × 40)

Typical histopathological features associated with obesity-related liver alterations include lobular and portal inflammation, hepatocyte ballooning, steatosis, apoptosis, and fibrosis [23]. Representative histological sections are shown in Fig. 6. According to the scoring system described in the Methods section, the obese control group (GC) was classified as grade 2, indicating hepatic involvement affecting approximately 33–66% of the tissue. In contrast, animals from the normal group (GN) did not exhibit histological lesions, and those supplemented with the carbohydrate-rich extract (T1) did not show evident histopathological alterations.

The reduction in adipose tissue observed in T1 animals may be associated with lower lipid flux to the liver, thereby reducing hepatic lipid accumulation and lipotoxicity in obesity-related conditions.

In this context, the histological findings, together with the biochemical and metabolic parameters evaluated, suggest an association between supplementation with the carbohydrate-rich extract and attenuation of obesity-associated hepatic alterations.

## Conclusion

This study highlights *Pereskia aculeata*, a leafy cactus native to Latin America, as a valuable source of functional carbohydrates. The carbohydrate-rich extract demonstrated in vitro prebiotic properties, including resistance to simulated gastrointestinal digestion, selective stimulation of beneficial lactic acid bacteria, and antioxidant activity in chemical assays. In the experimental obesity model, supplementation was associated with improvements in metabolic parameters, including fasting blood glucose and lipid profile, and with attenuation of hepatic alterations. These findings reinforce the potential of unconventional edible cacti from Latin American biodiversity as underexplored resources for nutrition and health applications. However, the prebiotic and antioxidant activities were not directly confirmed in vivo, and further studies are required to elucidate the mechanisms and functional effects of microbiota-mediated effects under physiological conditions.

## Supplementary Information

Below is the link to the electronic supplementary material.

Supplementary Material 1 (DOCX 39.1 KB)

Supplementary Material 2 (DOCX 44.9 KB)

## Data Availability

No datasets were generated or analysed during the current study.
