# Supplementary material for: Carbohydrate-Rich Extract from Pereskia aculeata Leaves: In Vitro Prebiotic-Related Properties and Metabolic Effects in an Experimental Model of Obesity
Source: Plant Foods Hum Nutr. 2026 Mar 21;81(2):36. doi: 10.1007/s11130-026-01486-0 (PMC13005867; doi:10.1007/s11130-026-01486-0)
Supplement: Supplementary file 1 — Supplementary Material 1 (DOCX 39.1 KB) [file 11130_2026_1486_MOESM1_ESM.docx]

**Carbohydrate-rich extract from *Pereskia aculeata* leaves: *in vitro* prebiotic-related properties and metabolic effects in an experimental model of obesity**

Martha Eunice de Bessa^1^, Vivian Tomasco Andrade^1^, Gabriele Moreira Guimarães^2^, Ana Flávia Lawall Werneck Cerqueira^3^, Carolina Carvalho Ramos Viana^4^,^5^, Marianna Miranda Furtado^6^, Renata de Freitas Mendes^1^, Mirian Pereira Rodarte^3^, Anderson S. Sant’Ana^6^, Maria José Valenzuela Bell^4^, Maria Christina Marques Nogueira Castañon^7^, Maria Silvana Alves^3^, Elisabeth Neumann^2^, Elita Scio^1^

^1^ Laboratory of Bioactive Natural Products, Department of Biochemistry, Biological Sciences Institute, Federal University of Juiz de Fora, 36.036-900, Juiz de Fora, MG, Brazil.

^2^ Department of Microbiology, Biological Sciences Institute, Federal University of Minas Gerais, Minas Gerais, 31270-901, Belo Horizonte, Brazil.

^3^ Department of Pharmaceutical Sciences, Faculty of Pharmacy, Federal University of Juiz de Fora, 36.036-900, Juiz de Fora, MG, Brazil.

^4^ Department of Physics, Institute of Exact Sciences, Federal University of Juiz de Fora, Juiz de Fora, MG, 36036-900, Brazil.

^5^ Cândido Tostes Dairy Institute, Juiz de Fora, MG, 36045-560, Brazil.

^6^ Department of Food Science and Nutrition, Faculty of Food Engineering, University of Campinas, Campinas, SP, 13083-862, Brazil.

^7^ Department of Morphology, Institute of Biological Sciences, Federal University of Juiz de Fora, Juiz de Fora, MG, 36036-900, Brazil.

Corresponding author: Scio, Elita email address: [elita.scio@ufjf.br](mailto:elita.scio@ufjf.br)

**Plant Foods for Human Nutrition**

**Materials and Methods**

**Plant Material and Extract Preparation**

Fresh leaves were dried at approximately 35 °C for 15 days and then ground to a fine powder. Approximately 1 kg of dried material was defatted by static maceration in 96° GL (1.5 L) ethanol for 78 h. After filtration, the residual plant material was resuspended in distilled water (1:15, w/v) and heated at 80 °C for 2 h. This extraction step was repeated for three consecutive cycles to remove residual ethanol and enhance polysaccharide extraction. The extract was vacuum filtered to separate the mucilaginous fraction, and polysaccharides were precipitated by gradual addition of 96° GL ethanol (final ethanol concentration or volume ratio: 500 mL, 3 volumes, v/v). The precipitation was centrifuged at 5.000 rpm for 20 min, concentrated by rotary evaporation at 50 °C, lyophilized, and stored in amber glass bottles at 5 °C.

**Proximate Composition and Phytochemical Screening**

The centesimal composition of *Pereskia aculeata* Miller leaves and the carbohydrate-rich extract was determined by: moisture content was determined by oven drying at 105 °C until constant weight. Ash content was obtained by incineration at 500 °C. Lipids were quantified by *petroleum* ether extraction using a Soxhlet apparatus, and protein content was determined by the Kjeldahl method using a nitrogen-to-protein conversion factor of 6.25. Total carbohydrates were calculated by difference. Reducing sugars, including glucose and fructose, were quantified as follows: An aliquot (1.0 mL) of the diluted sample was mixed with 1.0 mL of Somogyi's alkaline copper reagent and heated to 100 °C for 10 min. After cooling, 1.0 mL of Nelson's arsenomolybdate reagent was added and the volume was adjusted to 10 mL with distilled water before measuring the absorbance at 520 nm [12]. For this specific comparison (two independent groups), the data were analyzed using Student's t-test (independent samples), considering p < 0.05 as statistically significant. All physicochemical analyses were performed in triplicate, and the results are expressed as mean ± standard deviation. Preliminary phytochemical screening of the main classes of secondary metabolites was performed following the procedures described by Matos (1997) [13].

***In vitro* Antioxidant Activity**

Antioxidant activity was evaluated using different assays. Gallic acid and ascorbic acid were used as positive controls, and inulin was included for comparison. The tests were performed in triplicate.

Free radical scavenging activity was assessed using the DPPH, samples were tested at concentrations ranging from 0.98 to 250 µg/mL, and absorbance was measured at 517 nm after 30 min of reaction in the dark. Results were expressed as percentage inhibition of the DPPH radical.

Nitric oxide (NO) scavenging activity was determined using sodium nitroprusside as an NO donor, sample dilutions (7.87–250 µg/mL) were prepared in phosphate-buffered saline (pH 7.4), and nitrite formation was quantified using the Griess reagent at 540 nm.

Total antioxidant capacity was evaluated using the *phosphomolybdenum* reduction assay, samples were incubated with the *phosphomolybdenum* reagent at 95 °C for 90 min, and absorbance was measured at 695 nm. Results were expressed as percentage reduction of the *phosphomolybdenum* complex.

**Evaluation of Prebiotic Potential**

Growth assays were performed using *Lactobacillus acidophilus* NCFM, *Lacticaseibacillus casei* 25P, *Lactobacillus delbrueckii*, *Limosilactobacillus fermentum* ATCC 14931, *Lactiplantibacillus plantarum* ATCC 14917, and *Limosilactobacillus reuteri* 1/2Z. Strains were activated in MRS broth and cultivated at 37 °C. Before the experiments, the bacteria were activated by two passages in MRS broth and maintained aerobically for 24 hours at 37 °C. Bacterial growth was evaluated in a culture medium based on the standard formulation of De Man, Rogosa and Sharpe (MRS). For comparison, an MRS medium incorporated with a carbohydrate extract was prepared, replacing dextrose in the same proportion used in the standard medium [18]. The media were prepared with the pH adjusted to 6.8 and the cultures were incubated in an incubator at 37 °C for 24 hours. The growth test was performed at intervals of 0, 1, 2, 3, 4, 5, 6, 8, 10, 12 and 24 hours. For this, successive dilutions of the LAB samples were made, and 10 µL aliquots were plated on MRS agar using the drop plate technique for total cell counting and elaboration of the bacterial growth curve. Simultaneously, the density of the microbial population was verified by measuring the optical density at 600 nm using a spectrophotometer.

Antagonistic activity against *Salmonella enterica* subsp*. enterica* serovar Typhimurium *ATCC 14028*, and *Escherichia coli* ATCC 25723 was assessed using a spot-on-lawn assay, following Annuk et al. [19]. Effective protection halos were measured after incubation.

Simulated gastrointestinal digestion was conducted using a static in vitro system reproducing gastric, duodenal, and jejunal conditions. A static in vitro gastrointestinal digestion model was employed to simulate human upper gastrointestinal conditions. The protocol included sequential oral, gastric, and intestinal phases with controlled pH transitions to reproduce physiological conditions (oral phase pH 7.0; gastric phase pH 2.5–3.0; intestinal phase pH 7.0). All digestion steps were conducted at 37 °C under constant agitation to mimic body temperature and gastrointestinal motility. Incubation times were defined to reflect human digestive transit (oral phase: 2 min; gastric phase: 2 h; intestinal phase: 2 h). Enzymatic digestion was performed using standard enzymes (Sigma®-Aldrich, USA), including α-amylase for the oral phase, pepsin for the gastric phase, and pancreatin with bile salts for the intestinal phase. Enzyme activities and concentrations were adjusted according to established food digestion protocols to approximate physiological enzymatic conditions [20].

Samples were collected at the end of each phase for further analysis. Samples collected at each digestion stage were lyophilized and analyzed by FTIR-MIR to assess the stability of the extracts under gastrointestinal conditions.

**Experimental Animals and Obesity Model**

**Obesity model**

Male Wistar rats (21 days old, 25–35 g) were obtained from the Center for Reproductive Biology (UFJF). All procedures were approved by the Institutional Ethics Committee (protocol No. 01/2018). Animals were maintained with free access to water and a commercial standard diet (Nuvital®TM, Colombo, PR, Brazil) throughout the experimental period.

Obesity was induced using the litter-size reduction model described by Rinaldi et al. [21]. During the mating period, females were monitored and, upon confirmation of pregnancy, housed individually. Litters with 9 to 12 pups were considered normal (normal litter). On the third postnatal day, litter size was adjusted, and experimental litters were reduced to 3 male pups per litter (reduced litter). This early reduction induces metabolic reprogramming leading to overfeeding, and previous studies have demonstrated that this model results in obesity and hyperglycemia in Wistar rats [21].

Animals were allocated into three groups (n = 10 per group): normal litter (GN), reduced litter control (GC), and reduced litter supplemented with the carbohydrate-rich extract (T1).

After a one-week adaptation period, the T1 group received the extract diluted in drinking water (0.3% w/v, ad libitum) for 75 days, corresponding to an estimated intake of approximately 300 mg/kg body weight/day, based on average daily water consumption. The dose of the carbohydrate-rich extract used for supplementation in group T1 was defined based on the usual concentration range described for inulin [22], a well-established prebiotic used as a reference compound in experimental models. Body weight and naso-anal length were monitored throughout the experiment.

At the end of the protocol, the animals were euthanized by intraperitoneal administration of ketamine hydrochloride (90 mg/kg) combined with xylazine hydrochloride (10 mg/kg), following established anesthetic protocols for rodents. Blood samples were collected, and liver and adipose tissues were harvested for analysis.

**Biochemical and Histopathological Analyses**

Oral glucose tolerance tests were performed at 96 days of age, via gavage. After a 6-hour fasting period, animals were individually allocated in cages for the test. Baseline blood glucose (T0) was measured from a drop of blood obtained by tail tip incision and applied to reactive test strips (Accu-Chek Active®, Roche®), using a portable glucometer from the same manufacturer.

Immediately after baseline measurement, a glucose solution (dextrose, 2 g/kg body weight) was administered by oral gavage. Additional blood samples were collected from the tail at 30 (T1), 60 (T2), 90 (T3), and 120 (T4) minutes post-administration, and glucose levels were measured using the same device and procedure.

Serum biochemical parameters, including glucose, creatinine, ALT, AST, total cholesterol, LDL, HDL, and triglycerides, were determined using commercial diagnostic kits (Roche®), validated for biochemical analysis in Wistar rat samples and compatible with the Cobas C111 automated analyzer.

For histopathological analysis, a portion of the liver was collected and placed in a 10% formalin solution. The samples were distributed into cassettes. Subsequently, they were dehydrated in increasing concentrations of alcohol, cleared in two xylene exchanges, and embedded in paraffin. The prepared blocks were subjected to microtomy to obtain 4 µm thick sections using a manual rotary microtome. Next, the slides were sectioned with hematoxylin-eosin. The histological sections obtained from the paraffin blocks were deparaffinized in two xylene baths for 15 minutes each, hydrated in decreasing concentration solutions for 3 minutes each, and subsequently hydrated in running water for 20 minutes. Then, the sections were cut with hematoxylin for 50 seconds, washed in running water for 20 minutes, and washed with eosin for 1 minute and 30 seconds. Finally, the slide with the cut sections was mounted using Entellan® and a coverslip.

The analysis was performed by two analysts and using an optical criterion coupled to a digital camera. The analysis of the liver tissue was performed using the established criteria [23]. In summary, the score for steatosis was determined according to the percentage of liver parenchyma occupied by fat (0 = <5%, 1 = 5%-33%, 2 = 33%-66% e 3 = ˃ 66%).
