# Supplementary material for: Carbohydrate-Rich Extract from Pereskia aculeata Leaves: In Vitro Prebiotic-Related Properties and Metabolic Effects in an Experimental Model of Obesity
Source: Plant Foods Hum Nutr. 2026 Mar 21;81(2):36. doi: 10.1007/s11130-026-01486-0 (PMC13005867; doi:10.1007/s11130-026-01486-0)
Supplement: Supplementary file 2 — Supplementary Material 2 (DOCX 44.9 KB) [file 11130_2026_1486_MOESM2_ESM.docx]

**Carbohydrate-rich extract from *Pereskia aculeata* leaves: *in vitro* prebiotic-related properties and metabolic effects in an experimental model of obesity**

Martha Eunice de Bessa^1^, Vivian Tomasco Andrade^1^, Gabriele Moreira Guimarães^2^, Ana Flávia Lawall Werneck Cerqueira^3^, Carolina Carvalho Ramos Viana^4^,^5^, Marianna Miranda Furtado^6^, Renata de Freitas Mendes^1^, Mirian Pereira Rodarte^3^, Anderson S. Sant’Ana^6^, Maria José Valenzuela Bell^4^, Maria Christina Marques Nogueira Castañon^7^, Maria Silvana Alves^3^, Elisabeth Neumann^2^, Elita Scio^1^

^1^ Laboratory of Bioactive Natural Products, Department of Biochemistry, Biological Sciences Institute, Federal University of Juiz de Fora, 36.036-900, Juiz de Fora, MG, Brazil.

^2^ Department of Microbiology, Biological Sciences Institute, Federal University of Minas Gerais, Minas Gerais, 31270-901, Belo Horizonte, Brazil.

^3^ Department of Pharmaceutical Sciences, Faculty of Pharmacy, Federal University of Juiz de Fora, 36.036-900, Juiz de Fora, MG, Brazil.

^4^ Department of Physics, Institute of Exact Sciences, Federal University of Juiz de Fora, Juiz de Fora, MG, 36036-900, Brazil.

^5^ Cândido Tostes Dairy Institute, Juiz de Fora, MG, 36045-560, Brazil.

^6^ Department of Food Science and Nutrition, Faculty of Food Engineering, University of Campinas, Campinas, SP, 13083-862, Brazil.

^7^ Department of Morphology, Institute of Biological Sciences, Federal University of Juiz de Fora, Juiz de Fora, MG, 36036-900, Brazil.

Corresponding author: Scio, Elita email address: [elita.scio@ufjf.br](mailto:elita.scio@ufjf.br)

**Plant Foods for Human Nutrition**

**Table 1** Proximate composition of dried Pereskia aculeata Mill. leaves and the carbohydrate-rich extract (g/100 g).

| Components | Leaf powder (%) | Extract (%) |
| --- | --- | --- |
| Proteins | 15.62 ± 0.05* | 3.16 ± 0.03* |
| Lipids | 4.31 ± 0.02* | 0.33 ± 0.10* |
| Ash | 14.07 ± 0.02* | 1.87 ± 0.01* |
| Moisture | 12.30 ± 0.13* | 4.62 ± 0.11* |
| Carbohydrates | 53.70 ± 0.13* | 90.02 ± 0.13* |
| Reducing sugars | 2.08 ± 0.01^a^ | 1.71 ± 0.01^a^ |

Data are expressed as mean ± SD. * indicates significant differences between leaf powder and carbohydrate-rich extract (Student’s t-test, independent samples; *p* < 0.05). Identical superscript letters indicate no significant difference between groups. Reducing sugars refer to sugars with free carbonyl groups quantified by the Somogyi–Nelson method and expressed as glucose equivalents.

**Table 2** Viable cell counts of lactic acid bacteria grown on MRS agar supplemented with the carbohydrate-rich extract and on standard MRS agar over a 0–24 h incubation period

| Lactic acid bacteria (log CFU.mL⁻¹) | | | | | |  |
| --- | --- | --- | --- | --- | --- | --- |
| Group 1 |  |  |  |  |  |  |
| Time (h) | EA | MA | EC | MC | Ede | MDe |
| 0 | 2.0 ± 0.07ᵃ | 1.2 ± 0.05ᵃ | 2.1 ± 0.14ᵃ | 2.1 ± 0.11ᵃ | 2.4 ± 0.09ᵃ | 2.0 ± 0.05ᵃ |
| 2 | 3.3 ± 0.05ᵃ | 1.4 ± 0.09ᵇ | 2.0 ± 0.08ᵃ | 2.4 ± 0.08ᵃ | 2.0 ± 0.11ᵃ | 2.1 ± 0.06ᵃ |
| 4 | 4.3 ± 0.07ᵃ | 2.0 ± 0.09ᵇ | 5.4 ± 0.05ᵃ | 2.9 ± 0.08ᵇ | 4.0 ± 0.08ᵃ | 2.3 ± 0.09ᵇ |
| 6 | 6.0 ± 0.02ᵃ | 2.3 ± 0.15ᵇ | 7.0 ± 0.05ᵃ | 3.4 ± 0.06ᵇ | 6.9 ± 0.05ᵃ | 2.4 ± 0.05ᵇ |
| 8 | 9.2 ± 0.04ᵃ | 2.8 ± 0.11ᵇ | 7.6 ± 0.06ᵃ | 3.8 ± 0.07ᵇ | 7.0 ± 0.15ᵃ | 2.6 ± 0.08ᵇ |
| 10 | 9.5 ± 0.06ᵃ | 3.0 ± 0.08ᵇ | 8.0 ± 0.05ᵃ | 4.0 ± 0.09ᵇ | 7.9 ± 0.07ᵃ | 2.8 ± 0.13ᵇ |
| 12 | 9.7 ± 0.04ᵃ | 3.0 ± 0.07ᵇ | 8.6 ± 0.15ᵃ | 4.0 ± 0.06ᵇ | 8.4 ± 0.06ᵃ | 4.4 ± 0.05ᵇ |
| 24 | 10.0 ± 0.04ᵃ | 4.0 ± 0.02ᵇ | 9.0 ± 0.05ᵃ | 4.3 ± 0.05ᵇ | 8.8 ± 0.05ᵃ | 4.0 ± 0.05ᵇ |
| Group 2 |  |  |  |  |  |  |
| Time (h) | EF | MF | ER | MR | EP | MP |
| 0 | 2.4 ± 0.07ᵃ | 2.3 ± 0.11ᵃ | 2.5 ± 0.07ᵃ | 2.2 ± 0.09ᵃ | 2.4 ± 0.13ᵃ | 2.4 ± 0.09ᵃ |
| 2 | 3.0 ± 0.05ᵃ | 2.4 ± 0.08ᵃ | 6.0 ± 0.08ᵃ | 2.6 ± 0.07ᵇ | 2.4 ± 0.05ᵃ | 2.0 ± 0.05ᵃ |
| 4 | 8.9 ± 0.07ᵃ | 2.6 ± 0.09ᵇ | 6.9 ± 0.07ᵃ | 3.0 ± 0.05ᵇ | 6.3 ± 0.02ᵃ | 2.7 ± 0.06ᵇ |
| 6 | 8.0 ± 0.05ᵃ | 3.4 ± 0.05ᵇ | 7.2 ± 0.05ᵃ | 4.0 ± 0.04ᵇ | 7.3 ± 0.06ᵃ | 3.7 ± 0.08ᵇ |
| 8 | 9.3 ± 0.08ᵃ | 3.5 ± 0.07ᵇ | 8.9 ± 0.05ᵃ | 3.8 ± 0.06ᵇ | 8.5 ± 0.04ᵃ | 3.7 ± 0.08ᵇ |
| 10 | 9.4 ± 0.05ᵃ | 5.0 ± 0.04ᵇ | 9.0 ± 0.07ᵃ | 4.3 ± 0.04ᵇ | 8.7 ± 0.05ᵃ | 4.5 ± 0.04ᵇ |
| 12 | 9.5 ± 0.05ᵃ | 6.7 ± 0.07ᵇ | 9.3 ± 0.05ᵃ | 4.4 ± 0.05ᵇ | 9.4 ± 0.06ᵃ | 6.0 ± 0.05ᵇ |
| 24 | 10.0 ± 0.05ᵃ | 7.2 ± 0.03ᵇ | 9.5 ± 0.04ᵃ | 4.5 ± 0.06ᵇ | 9.8 ± 0.05ᵃ | 6.8 ± 0.05ᵇ |

Viable cell counts of different lactic acid bacteria strains grown on standard MRS agar (M) and on MRS agar supplemented with the carbohydrate-rich extract (E). (Group 1) *Lactobacillus acidophilus* NCFM (EA/MA), *Lacticaseibacillus casei* 25P (EC/MC), *Lactobacillus delbrueckii* UFV-H2-b20 (EDe/MDe); (Group 2) *Limosilactobacillus fermentum* ATCC 14931 (EF/MF), *Limosilactobacillus reuteri* 1/2Z (ER/MR), and *Lactiplantibacillus plantarum* ATCC 14917 (EP/MP). Data are expressed as mean ± SD. Different lowercase letters on the same line indicate statistically significant differences between the carbohydrate-rich extract–enriched medium and the standard MRS medium at the same time point (Student’s t-test, independent samples; *p* < 0.05).

**Table 3** Effect of carbohydrate-rich extract -enriched medium on the antagonistic activity of lactic acid bacteria against pathogenic potential microorganisms

| *Lactobacillus acidophilus* NCFM | | |
| --- | --- | --- |
| Culture medium | *Salmonella enterica* serovar Typhimurium (mm) | *Escherichia coli* (mm) |
| Enriched MRS (EA) | 12.5 ± 0.64ᵃ | 6.0 ± 0.24ᵃ |
| Standard MRS (MA) | 1.0 ± 0.02ᵇ | 2.0 ± 0.05ᵇ |
| *Lactobacillus casei* 25P | | |
| Culture medium | *Salmonella enterica* serovar Typhimurium (mm) | *Escherichia coli* (mm) |
| Enriched MRS (EC) | 16.0 ± 1.50ᵃ | 11.0 ± 0.05ᵃ |
| Standard MRS (MC) | 2.0 ± 0.05ᵇ | 3.0 ± 0.04ᵇ |
| Controls | *Salmonella enterica* serovar Typhimurium (mm) | *Escherichia coli* (mm) |
| Enriched MRS without bacteria (NE) | 0.0 ± 0.0 | 0.0 ± 0.0 |
| Standard MRS without bacteria (NM) | 0.0 ± 0.0 | 0.0 ± 0.0 |

EA/EC: MRS medium enriched with carbohydrate-rich extract; MA/MC: Standard MRS medium. Data are expressed as mean ± SD. Different superscript lowercase letters within the same column and for the same bacterial strain indicate statistically significant differences between media (Student’s t-test, independent samples; *p* < 0.05).

### ****Table 4**** Body mass index (BMI), Lee index, and **obesity-related parameters of Wistar rats subjected to the litter size reduction model**

| Groups | Body Mass Index g/cm^2^ | Index of LEE g/cm | Weight (g) | Retroperitoneal fat (g) | Perigonadal fat (g) | Adiposity Index (%) |
| --- | --- | --- | --- | --- | --- | --- |
| GN | 0.52 ± 0.06^c^ | 0.280 ± 0.02^c^ | 290 ± 2.21^c^ | 2.9 ± 0.05^c^ | 3.8 ± 0.42^c^ | 2.3 ± 0.47^c^ |
| GC | 0.74 ± 0.04^a^ | 0.320 ± 0.04^a^ | 330 ± 4.21^a^ | 5.6 ± 0.42^a^ | 5.6 ± 3.16^a^ | 3.3 ± 0.31^a^ |
| T1 | 0.59 ± 0.09^b^ | 0.301 ± 0.05^b^ | 300 ± 2.19^b^ | 4.5 ± 0.15^b^ | 4.7 ± 1.84^b^ | 3.0 ± 0.05^b^ |

Values are expressed as mean ± SD. GN, normal group; GC, obese control group; T1, group supplemented with carbohydrate-rich extract. Different superscript lowercase letters within the same column indicate statistically significant differences among groups (one-way ANOVA followed by Tukey’s post hoc test; *p* < 0.05).

**Table 5** Evaluation of the lipid profile of Wistar rats subjected to the litter reduction model

| Groups | Cholesterol mg/dL | Low-density lipoprotein - cholesterol mg/dL | High-density lipoprotein cholesterol mg/dL | Triglycerides mg/dL |
| --- | --- | --- | --- | --- |
| GN | 77 ± 1.19^b^ | 9 ± 1.19^b^ | 83 ± 2.19^a^ | 47 ± 2.01^b^ |
| GC | 120 ± 2.09^a^ | 15 ± 2.88^a^ | 65 ± 1.38^b^ | 88 ± 2.77^a^ |
| T1 | 91 ± 1.14^b^ | 10 ± 3.26^b^ | 84± 1.8^a^ | 61 ± 2.55^b^ |

Values are expressed as mean ± SD. GN, normal group; GC, obese control group; T1, group supplemented with carbohydrate-rich extract. Different superscript lowercase letters within the same column indicate statistically significant differences among groups (one-way ANOVA followed by Tukey’s post hoc test; *p* < 0.05).
